# Evaluation of an interdisciplinary chronic pain program and predictors of readiness for change

**DOI:** 10.1080/24740527.2019.1582296

**Published:** 2019-04-12

**Authors:** Laura Katz, Lisa Patterson, Ramesh Zacharias

**Affiliations:** a Michael G. DeGroote Pain Clinic, Hamilton Health Sciences, McMaster University Medical Centre, Hamilton, Ontario, Canada; b Department of Psychiatry and Behavioural Neurosciences, McMaster University, Hamilton, Ontario, Canada; c Institute of Pain Research and Care, McMaster University, Hamilton, Ontario, Canada; d Department of Anesthesia, McMaster University, Hamilton, Ontario, Canada

**Keywords:** chronic pain, interdisciplinary treatment, pain management, biopsychosocial, coping

## Abstract

**Background**: One in five Canadians experience chronic pain, and interdisciplinary pain programs are well established as the gold standard of treatment. However, not all patients are ready to engage in interdisciplinary treatment for chronic pain.

**Aims**: The aims of this study were to (1) first demonstrate changes in patient-related outcomes after attending a publicly funded 8-week interdisciplinary pain program and (2) evaluate pain-related predictors of readiness for change.

**Methods**: The institution’s research ethics board approved this study. One hundred twenty-nine patients completed questionnaires on the first and last day of attending the program. Paired sample *t*-tests were utilized to evaluate the changes in patient-related outcomes after attending the program, and linear regressions were utilized to evaluate pain-related predictors of the stages of change.

**Results**: Postprogram, there were significant decreases in pain-related interference, fear of pain/re-injury, pain catastrophizing, and symptoms of stress, depression, and anxiety and a significant increase in wellness-focused coping and self-efficacy. Postprogram, patients also demonstrated lower scores in precontemplation and contemplation and higher scores in action and maintenance stages of readiness for change. In predicting precontemplation, fear of pain/re-injury was the sole predictor, and self-efficacy was the sole predictor of the contemplation, action, and maintenance stages.

**Conclusion**: These results demonstrate the short-term benefits of an 8-week interdisciplinary pain program. It is suggested that preprogram interventions targeting kinesophobia for individuals who are precontemplative and self-efficacy for others may be important to facilitate patient engagement.

## RÉSUMÉ

**Contexte**: Un Canadien sur cinq souffre de douleur chronique. Il est bien établi que les programmes interdisciplinaires de la douleur constituent le traitement de référence. Toutefois, ce ne sont pas tous les patients qui sont prêts à suivre un traitement interdisciplinaire pour la douleur chronique.

**Buts**: Les buts de cette étude étaient : 1) démontrer les changements dans les résultats liés aux patients après que ces derniers aient participé à un programme interdisciplinaire de la douleur de huit semaines, financé par des fonds publics, et 2) évaluer les prédicteurs de la disposition au changement lié à la douleur.

**Méthodes**: La commission d’éthique de la recherche de l’institution a approuvé cette étude. Cent vingt-neuf patients ont répondu à des questionnaires le premier et le dernier jour de leur participation au programme. Des tests T pour échantillons appariés ont été utilisés pour évaluer les changements dans les résultats liés aux patients après avoir participé au programme et des régressions linéaires ont été utilisées pour évaluer les prédicteurs des étapes du changement lié à la douleur.

**Résultats**: Après le programme, des diminutions significatives ont été observées dans l’interférence liée à la douleur, la peur de la douleur ou d’une nouvelle blessure, la catastrophisation de la douleur, ainsi que dans les symptômes de stress, de dépression, et d’anxiété. Une augmentation significative des stratégies d’adaptatation axées sur le bien-être et l’efficacité personnelle a aussi été observée. Après le progamme, les patients ont aussi obtenu des scores plus faibles pour la pré-contemplation et la contemplation et des scores plus élevés pour les étapes d’action et de mantien de la disposition au changement. La peur de la douleur ou d’une nouvelle blessure était l’unique prédicteur pour la pré-contemplation et l’efficacité personnelle était l’unique prédicteur des étapes de la contemplation, de l’action et du maintien.

**Conclusion**: Ces résultats démontrent les bienfaits à court terme d’un programme interdisciplinaire de la douleur de huit semaines. Il est suggéré que les interventions pré-programme ciblant la kinésophobie, pour les individus qui sont à l’étape de la pré-contemplation, et l’efficacité personnelle pour d’autres, peuvent être importants pour faciliter l’engagement des patients.

## Introduction

One in five Canadians experience chronic pain, which imposes a significant burden on patients, caregivers, and the health care system and has a large personal, social, and economic impacts. Chronic pain is challenging to treat and unfortunately is frequently undertreated and undermanaged within Canada.^1,2^ Most treatments to date focus on the biomedical aspects of pain; however, chronic pain is a condition with biological, psychological, and social components.^3^ As such, the gold standard of treatment for chronic pain is from a biopsychosocial framework and an interdisciplinary perspective. The biopsychosocial approach takes into account the complex interaction between physiological, psychological, and social factors that influence one’s experience of chronic pain.^4^ Furthermore, group interdisciplinary treatments for chronic pain (including the professions of medicine, nursing, occupational therapy, physical therapy, and psychology) have been well established for their efficacy, cost-effectiveness, and longitudinal effects.^4–7^

In 2015, the Ministry of Health and Long Term Care in Ontario, Canada, provided funding for a number of pain clinics to develop interdisciplinary treatment programs for chronic pain. Such treatment programs aim to assist individuals in adopting a self-management approach to cope with their pain, emphasizing modifying unrealistic and negative thinking and increasing activity and productive functioning. Cognitive–behavioral therapy–focused programs are effective in reducing symptoms of pain; pain-related interference; and symptoms of depression, anxiety, and stress and improving functioning and active coping.^3,4^ Interdisciplinary pain programs may also utilize additional therapeutic modalities, such as acceptance and commitment therapy and mindfulness-based interventions, which are associated with improvements in physical and mental health in patients with chronic pain.^8^

Though interdisciplinary treatment programs are effective in the treatment of chronic pain, not all individuals are successful in engaging with and participating in such programs.^9^ The issue of high levels of attrition can be problematic, especially in publicly funded chronic pain management programs. Evaluating factors that are associated with understanding which patients engage and benefit from the programming while others do not is warranted due to both limited resources within a publicly funded system as well as burden on the patient in terms of time and effort. Evaluating the stage of change that an individual is in at the present time is one way of assessing for how ready he or she might be to engage in this approach to treatment.

The transtheoretical model of behavioral change suggests that individuals are in different stages of preparedness to make active behavioral changes.^10^ Readiness for change is divided into four stages, including precontemplation, contemplation, action, and maintenance. In the precontemplation stage of change, individuals express little interest in changing specific behaviors, and in the contemplation stage individuals report thinking about the possibility of making specific behavioral changes but are unlikely to make those changes soon. In the action stage, individuals actively take steps to change their behavior. Lastly, in the maintenance stage, individuals work to maintain health-related changes that they have made.^10^ Kerns et al. adapted these stages to individuals with chronic pain in the development of the Pain Stages of Change Questionnaire (PSOCQ).^9^ They further added to the stages in which individuals in the precontemplative stage of change continue to seek a medical cure for their pain and demonstrate reliance on passive coping strategies. Additionally, individuals in the maintenance stage are thought to have more self-control and accommodation of their pain and use more active coping strategies.

Research has demonstrated that measuring a patient’s stage of readiness for change may be useful in making treatment more efficient.^11^ Kerns and colleagues suggest that cognitive therapy strategies, such as cognitive restructuring, might be more appropriate for individuals in the precontemplative and contemplative stages of change, whereas behavioral strategies requiring more active coping might be more appropriate for individuals in the action and maintenance stages of change.^9^ Evaluating pain-related predictors of patients who are in various stages of change has the potential to increase efficiency of programs with limited resources and to provide potential targets for pre-intervention depending on which stage a patient is in. For example, a patient who is in the precontemplation stage of change and is enrolled in an interdisciplinary pain program may not be ready for such an approach, which might lead to suboptimal outcomes such as poor attendance, not engaging in the components of the program, or being disruptive to other patients and/or resistant during the program. As such, a targeted pretreatment intervention might help prepare the patient to better engage in the programming.

Literature evaluating patient-related factors that are associated with the stages of change is limited. Research has demonstrated that the stage of precontemplation is associated with a low intention to self-manage pain, higher pain-related interference, depression and pain-related anxiety, and beliefs that others/chance control their pain.^12,13^ Moreover, patients with higher precontemplation scores are less likely to complete treatment.^11,14^ Kerns and colleagues attempted to identify patients with chronic pain based on profiles of subscale scores on the PSOCQ and, as predicted, these patients did not differ in terms of measures of pain, disability, or demographics.^15^ For the purposes of clinical utility, this study was interested in evaluating pain-related variables that could be subject to modification with brief treatment in an interdisciplinary setting. Research has demonstrated that variables such as pain catastrophizing, kinesiophobia, and pain-related self-efficacy are modifiable with brief intervention.^16–19^

This article had two objectives: to (1) first demonstrate the changes in patient-related outcomes after attending the 8-week interdisciplinary pain program (in keeping with the Initiative on Methods, Measurement, and Pain Assessment in Clinical Trials recommendations)^20^ and (2) evaluate pain-related predictors on the PSOCQ to examine key factors that might lead to improving treatment and its efficacy. Addressing pain interference pre-intervention factors that predict a patient’s stage of change can assist in determining who will benefit from programming and be able to make improvements.

## Materials and methods

### Procedure

The institution’s research ethics board approved this study. Data were collected from patients who attended an 8-week interdisciplinary pain program at the Michael G. DeGroote Pain Clinic in Hamilton, Ontario, between March 2017 and July 2018 and consented to being a part of the study. Patients attending the clinic have a variety of chronic pain conditions. Patients can access the program through a referral from their family physician or from a physician at the Michael G. DeGroote Pain Clinic. All patients referred must attend an orientation session about the program

#### The 8-week interdisciplinary pain program

Patients initially attend an orientation session about the program and, if interested, are scheduled for an allied health and fitness assessment. If assessed to be appropriate for the program (e.g., no falls risk, interested in attending the program), patients attend the program once a week for 8 weeks for approximately 3 h each day. Each day consists of three components, including fitness/activation, a psychoeducation class, and relaxation. The psychoeducational classes include topics such as the science of chronic pain, dealing with flare-ups, pacing, activity education, sleep, nutrition, self-talk, and communication. Interdisciplinary team members include nurse practitioners, occupational therapists, pharmacists, physiotherapists, psychologists, psychometrists, and social workers who lead the classes. After consent was obtained, patients completed questionnaires on the first and last days of the program. Questionnaires were administered on the first day to collect baseline measurements before patients attended the programming and on the last day to be able to evaluate whether the programming was associated with any changes from baseline.

### Participants

This sample consisted of 129 adult patients who completed the 8-week interdisciplinary pain program, could read and write in English, consented to be a part of this study, and completed both admission and discharge questionnaire packages. Of those people, 80% (*n* = 102) completed at least five classes, 66.95% (*n* = 86) completed at least six classes, 49.15% (*n* = 63) completed at least seven classes, and 19.49 (*n* = 25) completed all eight classes. Briefly, patients were a mean of 49.88 ± 13.55 years old, had experienced pain for 12.88 ± 13.35 years, and were off work for 7.35 ± 6.47 years. Moreover, the majority of the patients were female (73.6%) and Caucasian (62.0%). Sample demographics are displayed in Table 1.

**Table 1.** Sample demographics.

	Years ± SD
Age	49.88 ± 13.55
Time since pain	12.88 ± 13.35
Time since work	7.35 ± 6.47
	% (*N*)
Place of birth—Canada	72.1 (93)
Gender—Female	73.6 (95)
Employment status	
Employed	23.3 (30)
Unemployed	19.4 (25)
Retired	18.6 (24)
Homemaker	1.6 (2)
Student	1.6 (2)
Ethnicity	
Caucasian	62.0 (80)
Asian	0.8 (1)
Black	3.9 (5)
Latin American	0.8 (1)
West/East Indian	3.1 (4)
First Nations	3.1 (4)
Education	
Elementary	1.6 (2)
High school	26.4 (34)
Some college/university	16.3 (21)
Graduate college/university	34.1 (44)
Postgraduate degree	7.8 (10)

### Measures

#### Demographics

Patients provided demographic information such as age, gender, ethnicity, place of birth, education level, employment status, time since pain problem began, and time since being off work.

#### Pain Intensity Scale

Pain intensity was assessed using a visual analog scale measuring patients’ “usual” and “least” amount of pain experienced in the past 2 weeks on a scale from 0 (*no pain*) to 10 (*unbearable pain*).^21^ This scale is a valid measure of assessing for pain intensity and highly reliable in relation to visual and verbal measures, with high responsiveness to change.^22,23^

#### Depression, anxiety, and stress

The Depression Anxiety and Stress Scales (DASS-21)^24^ was utilized to assess for symptoms of depression, anxiety, and stress, and asked patients to rate each statement on a scale from 0 (*did not apply to me at all*) to 3 (*applied to me very much, or most of the time*) in the past week. The DASS-21 is a valid measure of depression, anxiety, and stress in both nonclinical samples and clinical samples such as those with chronic pain.^24,25^

#### Pain catastrophizing

Catastrophizing was assessed using the Pain Catastrophizing Scale (PCS).^26^ The PCS asked patients to rate 13 statements describing different thoughts and feelings that may be associated with pain. Each item is scored on a Likert scale ranging from 0 (*not at all*) to 4 (*all the time*), with higher scores indicating higher levels of pain catastrophizing. The PCS is a well-established measure that is valid and reliable.^27^

#### Fear of pain/re-injury

Fear of pain/re-injury was assessed using the Tampa Scale of Kinesiophobia (TSK).^28^ Patients were asked to rate 11 items about how they feel about their pain on a scale from 1 (*strongly disagree*) to 4 (*strongly* agree). The TSK is a brief, reliable, and valid measure of fear of movement/re-injury for patients with chronic pain.^29^

#### Pain-related interference

Pain-related interference was assessed using the Pain Disability Index (PDI),^30^ which is a seven-item measure that assesses a patient’s degree of interference within seven life domains (i.e., family/home responsibilities, recreation, social activity, occupation, sexual behavior, self-care, life-support activity). Patients indicated their level of pain-related interference from 0 (*no disability*) to 10 (*total disability*). The PDI has good internal consistency and validity within chronic pain populations.^31^

#### Coping

The Brief–Chronic Pain Coping Inventory (B-CPCI) was used to measure behavioral coping.^32^ The B-CPCI consisted of 16 items, which asked patients to rate the frequency of use of behavioral and cognitive coping strategies. The items were grouped into the following eight subscales: Guarding, Resting, Asking for Assistance, Relaxation, Task Persistence, Exercising/Stretching, Seeking Social Support, and Coping Self-Statements. The frequency of these coping strategies was measured by the total number of days during which the strategy was used in the past week (0–7 days). Previous studies have demonstrated that the B-CPCI has good internal consistency, test–retest reliability, and significant correlations in the expected direction with measures of patient functioning.^33^ The illness-focused Coping subscale was calculated by averaging the items for Guarding, Resting, and Asking for Assistance, and the wellness-focused Coping subscale was calculated by averaging the items for Relaxation, Task Persistence, Exercising/Stretching, Seeking Social Support, and Coping Self-Statements.^34^

#### Readiness for change

The PSOCQ was utilized to assess patients’ readiness for change.^9^ Patients rated 30 statements on a scale from 1 (*strongly disagree*) to 5 (*strongly agree*), and the items were used to calculate four stages of change, including precontemplation, contemplation, action, and maintenance. The PSOCQ is a valid and reliable measure in patients with chronic pain.^9^

#### Self-efficacy

The Pain Self-Efficacy Questionnaire (PSEQ) was utilized to assess patients’ confidence in being able to perform a behavior/task despite experiencing pain and consisted of ten items rated on a scale from 0 (*not at all confident*) to 10 (*completely confident*).^35^ The PSEQ has strong psychometric properties, including high validity, reliability, consistency, and stability over time.^36^

### Data analysis

Data were analyzed using the statistical program SPSS version 25. Paired sample *t*-tests were employed to evaluate changes in outcomes pre- and postprogram. Linear regressions were also utilized to evaluate pain-related predictors at admission of readiness for change on the PSOCQ, which were entered simultaneously. The data were cleaned with respect to missing values. Data were found to be missing at random (*χ*^2^ = 169.23, *P* = 0.06), and data from questionnaires that were at least 85% complete were imputed using simple mean imputation. All other data were considered missing and the cases were deleted from the data set.

## Results

Correlations are listed in Table 2. Results from paired sample *t*-tests demonstrate significant improvements in patient-related outcomes postprogram (see Table 3). More specifically, there was a significant decrease in pain-related interference, fear of pain/re-injury, pain catastrophizing, and symptoms of stress, depression, and anxiety and a significant increase in wellness-focused coping and pain coping self-efficacy. Moreover, patients demonstrated lower scores in precontemplation and contemplation and higher scores in action and maintenance stages of readiness for change postprogram. The scores for pain intensity and illness-focused coping did not significantly change postprogram.

**Table 2.** Correlations.

	1	2	3	4	5	6	7	8
Pain	—							
TSK	0.26*	—						
PCS	0.37**	0.50**	—					
PSEQ	−0.40**	−0.24*	−0.47**	—				
Precontemplation	0.23*	0.54**	0.35**	−0.23*	—			
Contemplation	−0.08	−0.12	−0.04	0.27*	−0.24*	—		
Action	−0.15	−0.07	−0.25*	0.31**	−0.29**	0.32**	—	
Maintenance	−0.10	−0.13	−0.23*	0.34**	−0.18	0.13	0.59**	—

*Correlation is significant at the 0.05 level (two-tailed). **Correlation is significant at the 0.01 level (two-tailed). All values are from scores at admission.

Pain = average pain intensity, TSK = total score on the Tampa Scale of Kinesiophobia; PCS = total score on the Pain Catastrophizing Scale; PSEQ = total score on the Pain Self-Efficacy Questionnaire; Precontemplation, Contemplation, Action, and Maintenance = average subscale scores on the Pain Stages of Change Questionnaire.

**Table 3.** Paired sample *t*-tests comparing changes in outcomes pre- and postprogram.

	Preprogram			Postprogram						
	Mean	SD	Chronbach’s alpha	Mean	SD	Chronbach’s alpha	*t*	*P*	Effect size (Cohen’s *d*)	Range
Average pain	5.95	1.85	0.70	5.83	1.83	0.68	0.91	0.37	0.07	Small
Pain-related interference (PDI)	41.54	13.07	0.85	38.82	13.14	0.85	2.46	0.02*	0.21	Small
Fear of pain/re-injury (TSK)	29.62	6.78	0.86	27.06	6.78	0.86	3.88	<0.01**	0.38	Small
Pain catastrophizing (PCS)	24.69	12.30	0.95	20.96	13.04	0.95	4.01	<0.01**	0.29	Small
Illness-focused coping (CPCI)	4.62	1.46	0.70	4.37	1.42	0.67	1.77	0.08	0.18	Small
Wellness-focused coping (CPCI)	3.51	1.45	0.77	4.33	1.24	0.72	−6.08	<0.01**	0.61	Medium
Depression (DASS-21)	7.91	5.31	0.92	6.26	4.95	0.88	3.75	<0.01**	0.32	Small
Anxiety (DASS-21)	6.26	4.80	0.81	5.53	4.69	0.78	2.33	0.02*	0.15	Small
Stress (DASS-21)	10.24	5.22	0.88	8.61	4.69	0.84	3.33	<0.01**	0.33	Small
Precontemplation (PSOCQ)	2.69	0.73	0.78	2.38	0.68	0.77	4.45	<0.01**	0.44	Small
Contemplation (PSOCQ)	3.98	0.50	0.83	3.85	0.52	0.80	2.33	0.02*	0.24	Small
Action (PSOCQ)	3.55	0.58	0.70	4.00	0.59	0.82	−6.83	<0.01**	0.76	Medium
Maintenance (PSOCQ)	3.33	0.68	0.81	3.97	0.59	0.86	−8.09	<0.01**	0.99	Large
Pain self-efficacy (PSEQ)	26.54	12.28	0.90	32.00	12.56	0.92	−5.71	<0.01**	0.44	Small

*Correlation is significant at the 0.05 level (two-tailed). **Correlation is significant at the 0.01 level (two-tailed).

PDI = Pain Disability Index; TSK = Tampa Scale of Kinesiophobia; PCS = Pain Catastrophizing Scale; CPCI = Chronic Pain Coping Inventory; DASS-21 = Depression Anxiety and Stress Scale; PSOCQ = Pain Stages of Change Questionnaire; PSEQ = Pain Self-Efficacy Questionnaire.

Following are the results from the regressions evaluating the pain-related predictors of the stages of readiness for change. See Table 4 for regression statistics. In predicting the precontemplation stage of change, fear of pain/re-injury was the sole predictor. Moreover, in predicting the contemplation, action, and maintenance stages of change, pain self-efficacy was the sole predictor.

**Table 4.** Regressions predicting pain stages of change.

	Pain	TSK	PCS	PSEQ
Precontemplation (*F* = 4.80, *R*^2^* = *0.24, *P* < 0.01**)	*β *= −0.11, *P* = 0.36	*β *= 0.52, *P* < 0.01**	*β *= −0.20, *P* = 0.16	*β *= −0.21, *P* = 0.12
Contemplation (*F* = 2.66, *R*^2^* = *0.15, *P* = 0.04*)	*β *= 0.12, *P* = 0.39	*β *= −0.22, *P* = 0.13	*β *= 0.12, *P* = 0.43	*β *= −0.39, *P* < 0.01**
Action (*F* = 2.87, *R*^2^* = *0.17, *P* = 0.03*)	*β *= −0.04, *P* = 0.78	*β *= −0.16, *P* = 0.26	*β *= −0.14, *P* = 0.37	*β *= 0.34, *P* = 0.02*
Maintenance (*F* = 4.56, *R*^2^* = *0.24, *P* < 0.01**)	*β *= 0.04, *P* = 0.74	*β *= −0.09, *P* = 0.48	*β *= 0.03, *P* = 0.82	*β *= 0.49, *P* < 0.01**

*Regression is significant at the 0.05 level (two-tailed). **Regression is significant at the 0.01 level (two-tailed).

Pain = average pain intensity score; TSK = Tampa Scale of Kinesiophobia; PCS = Pain Catastrophizing Scale; PSEQ = Pain Self-Efficacy Questionnaire.

## Discussion

The aim of this study was to first demonstrate the changes in patient-related outcomes after attending the 8-week interdisciplinary pain program and then to evaluate pain-related predictors of readiness for change. Results from this study demonstrate that the 8-week interdisciplinary pain program was associated with significant improvements in pain-related interference; fear of pain/re-injury; pain catastrophizing; symptoms of depression, anxiety, and stress; and pain self-efficacy. Moreover, upon completion of the program, patients were more likely to be in the action and maintenance stages of change rather than the precontemplation and contemplation stages. Interestingly, patients did not report a change in their pain intensity postprogram. This is likely due to the fact that the goal of the program was to teach patients direct skills and strategies to better manage their pain and increase their functioning and not to decrease their intensity of pain experienced, consistent with other pain management programs in the literature.^3^ Though patients did not report a significant change in illness-focused coping, they did report a significant increase in wellness-focused coping postprogram.

In terms of the results from the analyses on pain-related predictors of readiness for change, fear of pain/re-injury was the sole predictor of the precontemplation stage of change, and pain self-efficacy was the sole predictor of the contemplation, action, and maintenance stages of change. Interestingly, pain intensity was not a significant predictor in any of the stages of change. These results demonstrate that patients entering a publicly funded interdisciplinary pain program were at different stages of readiness for change regardless of intensity of pain experienced and that stage may be predictive of how they engage with and participate in the programming. Patients who are in the precontemplative stage (meaning that they express little interest in changing specific behaviors and potentially believe that pain is a medical problem and should be managed by physicians and interventions) are more likely to have an increased fear of pain and re-injury, potentially leading to decreased engagement in the program and less readiness for change. Moreover, patients who are more ready to make changes (e.g., in the contemplative, action, or maintenance stage of change) and manage their pain in accordance with a biopsychosocial framework are more likely to experience higher pain coping self-efficacy.

These results may have some implications for potential preprogram interventions to better engage patients for improved participation. For example, if patients are primarily in the precontemplative stage of change, they might benefit from a preprogram intervention to decrease their fear of pain/re-injury. Patients in the precontemplative stage enrolled in an interdisciplinary pain management program that requires individuals to be active in learning, applying, and integrating coping skills and strategies are likely to be disengaged and might not benefit from the program at that point in time. This is burdensome to patients in terms of their time and efforts, as well as to the program with limited resources in a publicly funded system. There are a number of interventions to decrease kinesiophobia, such as psychoeducation and graded exposure, that might be of benefit as preprogram interventions to help move patients in the precontemplative stage further along the readiness for change continuum.^17,18^

Patients who are further along the continuum of readiness for change (e.g., contemplative, action, or maintenance stage of change) might benefit from additional support to increase their pain coping self-efficacy to enhance their ability to engage in learning and utilizing the skills and strategies to better cope with their pain. A 2016 systematic review posited that pain self-efficacy has the most empirical support for increasing treatment adherence in patients with chronic pain in interdisciplinary pain programs in comparison to other pain-related variables.^37^ Interventions to increase self-efficacy are effective in other clinical populations, such as people with addiction, and can range from verbal persuasion to experiential activities in formats such as group activities or computer-generated letters.^38^ In samples of patients with chronic pain, recent research has investigated whether the effects of a self-efficacy intervention can improve the outcomes in a sample of patients with chronic low back pain.^39^ Interventions aimed at increasing self-efficacy in patients with chronic pain can be provided as a brief booster on each day of program attendance or upon completion of the program as a strategy for relapse prevention.

There are a number of limitations of this study. First, patients who chose to attend the groups, consented to participate in this study, and completed questionnaires likely are a fairly motivated and engaged sample, which represents a potential sampling bias. However, due to the nature of this study, it was not possible to collect data on patients who did not consent to participating in the research. Future research could collect a larger sample of clinical data to determine the patient characteristics of individuals who are not interested in engaging with or participating in interdisciplinary pain programs. Moreover, the sample of this study was primarily female and Caucasian, which might lead to issues with generalizability and highlight potential issues of program accessibility and barriers to participation. Though the demographics of this sample were comparable to those of similar studies in the region,^40,41^ this is an important area for future research, especially in relation to publicly funded programs. Additionally, although the regression models suggest that pain-related outcomes predict the stages of change, this is simply an association and not an assumption of direction or causation. Furthermore, multiple tests to demonstrate postprogram outcomes were run in this study, which may result in incorrectly rejecting the null hypothesis. Moreover, the data linking the number of sessions that patients attended could not be connected to patient questionnaires to be able to associate this variable to the pain stages of change, which would be a valuable area for future research. Though the results from this study can provide valuable insights, future research would be helpful to evaluate these relationships longitudinally to determine whether pre-interventions do in fact help to shift patients along the readiness for change continuum and enhance engagement and to ascertain whether the changes found in the program are durable over time. Lastly, results from this study are based on one sample, and future research is needed in order to confirm these results in other samples and potentially within more specific patient populations.

## Conclusion

This study demonstrated that the 8-week interdisciplinary pain program is associated with improvements in patient-related outcomes. It also demonstrated that fear of pain/re-injury was predictive of being in the precontemplative stage of change, whereas pain self-efficacy was predictive of being in the contemplative, action, or maintenance stage of change. These results show the efficacy of a publicly funded interdisciplinary pain management program. They also suggest the importance of potential preprogram targeted interventions to decrease fear of pain/re-injury in some patients and to enhance coping self-efficacy in others for better patient engagement and efficiency of publicly funded programming.
